# Measuring Coverage in MNCH: Design, Implementation, and Interpretation Challenges Associated with Tracking Vaccination Coverage Using Household Surveys

**DOI:** 10.1371/journal.pmed.1001404

**Published:** 2013-05-07

**Authors:** Felicity T. Cutts, Hector S. Izurieta, Dale A. Rhoda

**Affiliations:** 1 Independent consultant, La Londe les Maures, France; 2 Food and Drug Administration, Rockville, Maryland, United States of America; 3 Battelle Memorial Institute, Columbus, Ohio, United States of America; Wellcome Trust Senior Research Fellow in Clinical Science, UCL Reader in International Child Health, Honorary Consultant, Great Ormond Street Hospital for Children, United Kingdom

## Abstract

Vaccination coverage is an important public health indicator that is measured using administrative reports and/or surveys. The measurement of vaccination coverage in low- and middle-income countries using surveys is susceptible to numerous challenges. These challenges include selection bias and information bias, which cannot be solved by increasing the sample size, and the precision of the coverage estimate, which is determined by the survey sample size and sampling method. Selection bias can result from an inaccurate sampling frame or inappropriate field procedures and, since populations likely to be missed in a vaccination coverage survey are also likely to be missed by vaccination teams, most often inflates coverage estimates. Importantly, the large multi-purpose household surveys that are often used to measure vaccination coverage have invested substantial effort to reduce selection bias. Information bias occurs when a child's vaccination status is misclassified due to mistakes on his or her vaccination record, in data transcription, in the way survey questions are presented, or in the guardian's recall of vaccination for children without a written record. There has been substantial reliance on the guardian's recall in recent surveys, and, worryingly, information bias may become more likely in the future as immunization schedules become more complex and variable. Finally, some surveys assess immunity directly using serological assays. Sero-surveys are important for assessing public health risk, but currently are unable to validate coverage estimates directly. To improve vaccination coverage estimates based on surveys, we recommend that recording tools and practices should be improved and that surveys should incorporate best practices for design, implementation, and analysis.

## Abstract 2

In a *PLOS Medicine* Review, Felicity Cutts and colleagues describe the challenges facing the estimation of vaccination coverage in low- and middle-income countries using surveys and recommend ways to improve the measurement of this important public health indicator.

*This paper is part of the* PLOS Medicine *“Measuring Coverage in MNCH” Collection.*

## Introduction

The percentage of a population that has been vaccinated—vaccination coverage—is an imperfect but helpful measure of the effectiveness of vaccination programs and of public health more broadly [1]. Vaccination coverage is a tracer condition for results-based financing [2], an indicator of eligibility for Millennium Challenge Account assistance [3], and a criterion for support from the GAVI Alliance for the introduction of new vaccines [4]. Making funding decisions contingent on coverage potentially incentivizes inflation of coverage figures, and there is wide recognition of the need to improve the data [5]–[8].

Ideally, vaccination coverage should be monitored continuously using registries or administrative reports [9]. Electronic immunization registries aim to document all vaccinations of each individual in each birth cohort [10],[11]. Denominators may derive from the same registry [11] or from a separate vital statistics system. When well implemented, electronic immunization registries can provide data for coverage measurement and for program management activities such as monitoring vaccine supply and requisitions and sending vaccination reminders. However, challenges facing such registries include accounting for migration within and between countries, ensuring complete birth registration and vaccination reporting, avoiding record duplication [12], and ensuring continuity after organizational changes [13]. Although pilot studies of electronic registries are ongoing in low- and middle-income countries including Albania, Guatemala, India, and Viet Nam [14], these challenges currently limit their use. Therefore, in most low- and middle-income countries, “administrative coverage” is calculated using aggregate reported data on the number of doses of each vaccine administered to children in the target age group in a given time period and target population estimates from censuses [1]. Health workers at each health facility typically compile data manually each month from clinic records such as immunization registers or tally sheets. At each vaccination visit, the health worker records vaccinations on clinic records and on a vaccination card, child health card, or other home-based record (HBR) that the mother keeps. The HBR serves as an educational tool for the mother and is also an important data source in household surveys. In many countries, however, the quality of primary recording of vaccinations, of transcription and compilation of data, and of reporting is low, and numerators may be either inflated (e.g., because doses outside the recommended age range are included) or too low (e.g., if private practitioners do not report). Moreover, denominators are often grossly inaccurate [5],[7]. Hence, wherever possible other data sources such as surveys are still considered in the World Health Organization (WHO)–United Nations Children's Fund (UNICEF) estimates of national immunization coverage [15].

Given current problems with coverage estimates based on administrative reports in many countries, we believe that surveys will continue to provide important information in the short-to-medium term, at national and sub-national levels. It is therefore critical that surveys are conducted rigorously. In this review, which is part of the *PLOS Medicine* “Measuring Coverage in MNCH” Collection, we discuss the survey methods used to estimate vaccination coverage in low- and middle-income countries, highlight potential pitfalls, and propose strategies to improve coverage measurement. Our review aims to inform public health practitioners and the researchers who design and implement surveys as well as Ministry of Health officials and donors who interpret and use data from surveys.

## Survey Methods Used to Measure Vaccination Coverage

Four types of surveys are commonly employed to estimate vaccination coverage (Table 1). The Demographic and Health Surveys (DHS) [16] and Multiple Indicator Cluster Surveys (MICS) [17] are probability sample surveys, in which each household has a known and nonzero probability of being selected in the sample. There have been about 10–15 DHS and 20 MICS per year since 1995. These large, important, and generally well-conducted household surveys, which are used to collect data about many aspects of health, are described in detail in a companion paper in this Collection [18].

**Table 1:** Characteristics of common surveys used to measure vaccination.

Survey Characteristic	DHS	MICS	EPI	LQAS
**Primary objectives**	Collection of information on a wide range of population, health, and nutrition topics, plus additional optional modules	Collection of information on population health, child protection, and child development	Estimation of vaccination coverage	Classification of lots (catchment areas) into two groups: those with adequate coverage and those with inadequate coverage
**Sampling scheme**	Stratified cluster sampling; clusters selected using PPES; clusters are usually census enumeration areas	Stratified cluster sampling; clusters selected using PPES; clusters are usually census enumeration areas	Cluster sampling with or without stratification; clusters are usually villages or urban neighborhoods, selected using PPES	Classic method uses simple random sampling within a lot; when lots are large, cluster sampling is sometimes employed
**Household selection**	Household selected randomly based on a complete household listing and mapping in the sample clusters	Current practice is random selection of households based on a complete listing and mapping of enumeration areas	Varies; usually non-probability; the first household is selected randomly, then neighboring households are selected until seven children can be enrolled	When cluster sampling is used, the first household is selected randomly before moving in a consistent direction, sampling every *k*th household
**Total sample size**	Based on desired precision for key indicators at the regional level; the number of children aged 12–23 months covered in recent surveys is typically around 1,800 at the national level	Based on desired precision of key indicators selected by implementing agencies; usually >2,000 women and several hundred children aged 12–23 months	Usually 30 clusters of seven children aged 12–23 months; sized to yield estimate of ±10% assuming design effect of two	Varies greatly; 19 respondents per lot is a common size with simple random sampling; 50 or 60 is common when using cluster sampling
**Respondents**	All men and women aged 15–49 years; vaccination data on children <5 years if biological mother is interviewed, and on women of childbearing age	All women aged 15–49 years; vaccination data on children <5 years if primary caretaker is interviewed, and on women of childbearing age	Mother or primary caretaker of children aged 12–23 months	Varies; field workers interview caretaker and when possible substantiate response with vaccination record or sometimes indelible ink finger mark on child
**Questionnaire length**	Household: 25 pages; woman's questionnaire: about 70 pages	Household: 18 pages; woman's: 38 pages; children under 5 years: 18 pages	1–2 pages	Often 1 page
**Implementers**	Usually National Statistical Office or equivalent, with capacity-building from MEASURE DHS	Usually National Statistical Office, with support from UNICEF and other partners	Varies; often national- or district- level Ministry of Health employees	Varies; usually independent from vaccination team
**Duration**	12 months or more to plan, implement, analyze, and report	12 months or more to plan, implement, analyze, and report	Several months to plan; weeks to implement, analyze, and report	Varies; 1–2 days per lot to implement and analyze

PPES, probability proportional to estimated population size.

The Expanded Programme on Immunization (EPI) cluster survey was developed by the WHO and was described in 1982 as a practical tool to quickly estimate coverage to within ±10 percentage points of the point estimate [19]. The original EPI survey method selects 30 clusters from which seven children in each cluster are selected using the “random start, systematic search” method. Specifically, a starting dwelling is chosen by starting at a central location in the village or town, selecting a direction at random, counting the dwellings lying in that direction up to the edge of the village, and selecting one of them randomly; adjacent households are then visited until seven children aged 12–23 months have been enrolled [20],[21]. The central starting location may bias the method to include households with good access to vaccination, so it is difficult to assign unbiased probabilities of selection to the households using this method, which does not meet the above criteria for a probability sample and is, therefore, a “non-probability sampling” survey method [22]. EPI surveys are widely used at national and sub-national levels, but there is no central database of results, so the total number of surveys conducted is unknown. Adaptations of the EPI survey have incorporated probability sampling at the final stage of sample selection [22]–[26], and the updated WHO guidelines [21] as well as a recent companion manual on hepatitis B immunization surveys emphasize the need for probability sampling for scientifically robust estimates of coverage [27].

The main design differences between EPI surveys (if probability sampling is used) and DHS or MICS surveys is that EPI surveys focus specifically on vaccination data while DHS and MICS surveys cover a wide range of population and health topics and include a much larger sample size. In addition, field implementation of EPI surveys is variable and often done without external technical assistance, while the DHS and MICS are highly standardized and have substantial technical assistance and quality control.

A final household survey method commonly used to estimate health intervention coverage in low- and middle-income countries is Lot Quality Assurance Sampling (LQAS). LQAS surveys use a stratified sampling approach to classify “lots,” which might be districts, health units, or catchment areas, as having either “adequate” or “inadequate” coverage of various public health interventions. For vaccination coverage measurement, LQAS is “nested” within a cluster survey to evaluate neonatal tetanus elimination [28], coverage of yellow fever vaccination [29], and coverage of meningococcal vaccine campaigns [30], and to monitor polio vaccination coverage after supplementary immunization activities [31].

## Survey Design and Implementation

Surveys used to estimate vaccination coverage should have a sample size that results in an acceptable sampling error, and implementation should minimize non-sampling errors, including selection bias and information bias (Table 2). In DHS and MICS surveys, the sample size is determined by the estimated number of households required for the desired precision of key indicators (not vaccination coverage), and all children in the eligible age groups in those households are included. In recent DHS surveys this design has given sample sizes of around 1,800 children aged 12–23 months. EPI surveys traditionally included 210 children aged 12–23 months in 30 clusters, but the sample size and number of clusters should be calculated according to assessments of the likely coverage, intra-cluster correlation, and desired precision of the vaccination coverage estimate [21],[27].

**Table 2:** Main potential sources of error and strategies to minimize them in population-based surveys measuring vaccination coverage.

Source of Error	Effect of Error on Results	Strategies to Minimize Error
**Random error**		
Sampling error	Reduces precision	Choose optimum sample design (e.g., number and size of clusters) and adjust sample size to achieve desired precision while retaining budgetary and logistical practicality
**Systematic error**		
Selection bias—sampling frame	Depends on size of excluded population and difference in vaccination uptake between those excluded and included	Use most recent census data available
		Assess likelihood of census projections reflecting reality and update census if necessary
		If large populations have been excluded (e.g., security constraints at time of census), consider special efforts to include them
Selection bias—sampling procedures	Non-probabilistic sampling may lead to bias in either direction	Use probability sampling method (plan time for listing of households within selected clusters)
		Use appropriate weighting in analysis
Selection bias—poor field procedures	Most likely to lead to upward bias in coverage results	Preselect households and ensure strict supervision
		Conduct survey at time of year and of day when people most likely to be available
		Work with communities to enhance survey participation rates
		Conduct revisits as necessary to locate caregivers and HBRs
		Do not substitute households
Information bias—lack of HBR or poorly filled HBR	Bias in coverage results may underestimate or overestimate coverage depending on how missing data are handled and how HBRs are read by enumerators	Public health programs need to educate families to retain HBRs and improve primary recording of vaccination data
		Publicize reminders about HBRs prior to survey (e.g., during household listing step)
		Allow time for mothers to look for HBR, revisit if necessary
		Include younger age groups in surveys and measure age-appropriate vaccination coverage
		Include questions as to condition of HBR and checks for errors
		Seek health facility–based records on children without HBR
Information bias—inaccurate verbal history	Most likely to bias infant coverage upwards as mothers may feel pressure to say their children have been vaccinated; for tetanus toxoid in adult women, verbal history usually underestimates percent of women protected	Ensure interviewers maintain neutral attitude
		Give time to mothers to respond
		Shorter questionnaires likely to have less interviewee fatigue
		Standardize questions, use visual aids, conduct close supervision
		For tetanus toxoid, ask careful questions about *all* doses received in previous and current pregnancies and in campaigns (but this still does not account for diphtheria-tetanus-pertussis vaccination received in infancy); sero-surveys play a useful role in the measurement of the prevalence of protection
Data transcription and data entry errors	May increase data classed as missing; can bias coverage results	Conduct close supervision
		Conduct range and consistency checks; enumerators can revisit household if necessary to correct data
Missing data	If nonrandom, biases result, often upwards	Conduct high-quality planning, training, and supervision to reduce missing data
		Include appropriate statistical adjustment for missing data

Selection bias may occur due to use of an outdated or nonrepresentative sampling frame, use of non-probability sampling, or poor field worker practices such as substituting a selected household with one that is easier to reach. The “random start, systematic search” method used in traditional EPI surveys has intrinsic geographic bias. It allows field workers to select households rather than this being part of the initial sampling process, does not document reasons for nonparticipation, and cannot adjust for biases resulting from out-of-date size estimates for selection of clusters using probability proportional to estimated size sampling. Moreover, in EPI and LQAS surveys, teams are likely to replace households where no one is home or where eligible respondents refuse to participate. If respondents are not selected randomly and if the same forces that influence participation in the survey also influence participation in vaccination (e.g., families missed by interviewers because they work in the fields all day may also lack time to attend vaccination clinics), replacement is likely to result in bias, probably upwards. Finally, surveys of the vaccination status of living individuals are inherently subject to selection bias since death is more likely in unvaccinated than in vaccinated children. In settings where there is a high infant mortality rate, this bias may be substantial.

**Figure 1:**
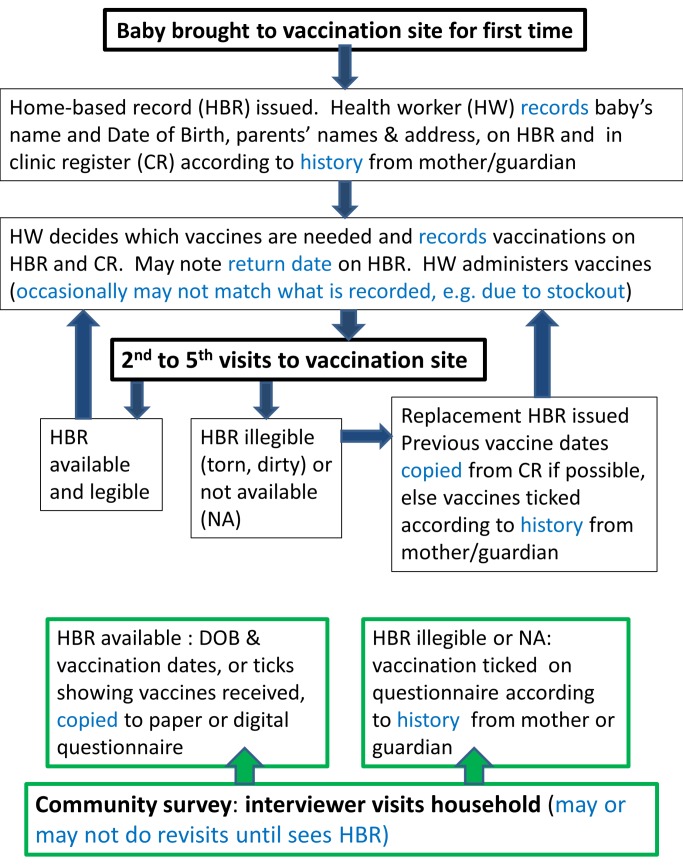
Schematic of recording of vaccination data at the time of vaccination and during community surveys. Recording at the time of vaccination (primary recording) is indicated in black boxes; recording during surveys is indicated in green boxes. Main potential sources of information error and bias are highlighted in blue. DOB, date of birth.

**Figure 2:**
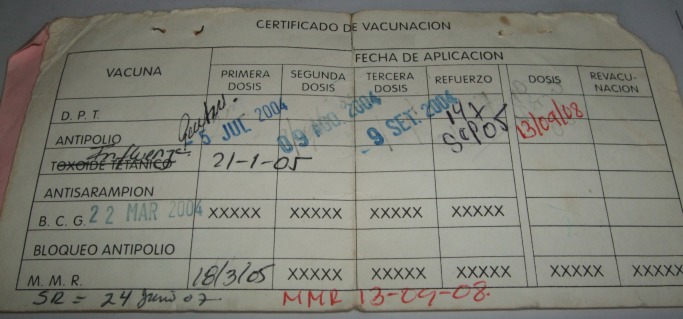
Several instances of improvisation on a vaccination card. (Photo courtesy of Carolina Danovaro, Pan American Health Organization.)

There are multiple potential sources of information error and bias in measuring the vaccination status of each child in surveys (Figure 1), many of which also affect data included in administrative reports. Mistakes can occur during primary data recording each time a child attends a vaccination point or when survey interviewers transcribe birth and vaccination dates onto a paper or digital questionnaire. If a paper questionnaire is used, further errors can occur during digital data entry. Data on source documents can also be incomplete [32] or inaccurate [33] and, when new vaccines are introduced, old HBRs may remain in circulation, requiring health workers to improvise in their recording (Figure 2). There is further confusion regarding recording of vaccines administered during campaigns such as “vaccination weeks” on HBRs [34].

When the HBR is not available (it may be lost or locked away, or mothers may not be given enough time to find it), interviewers question the child's parent or guardian to construct a verbal vaccination history. The reliability of such histories may vary with the information received or understood by mothers at the time of vaccination; the interviewer's skills, carefulness, neutral demeanor, and use of appropriate language; the recall period; and the length of the questionnaire and resulting interview fatigue [35]–[37]. The complexity of the vaccination schedule can also affect the reliability of a verbal vaccination history. When the EPI survey was introduced in the 1980s, the infant EPI schedule comprised five visits, which lent themselves to straightforward questions to the mother (Table 3). Because current schedules are much more complex (Table 4) and vary over time and between countries, constructing a verbal history of vaccinations received is now much more difficult and likely to become increasingly so. Thus, the questions included in surveys need substantial and continuous adaptation.

**Table 3:** Illustrative questions used in the past to elicit a verbal history of vaccination according to the EPI schedule in the 1980s.

Recommended Age for Vaccination	Vaccines and How Administered	Example Questions to Mother to Elicit Verbal History
Birth	BCG (intradermally, usually in the upper arm)	Did the child receive an injection in the upper arm soon after birth? (check for scar)
6 weeks	First dose of DTP (subcutaneous or intramuscular injection, usually in the thigh) and OPV (oral)	Did the child receive an injection in the thigh (the “triple vaccine”)? If yes, how many times? Did the child also receive drops in the mouth? If yes, how many times?
10 weeks	DTP, OPV 2	Same as for 6 weeks
14 weeks	DTP, OPV 3	Same as for 6 weeks
9 months	Measles (subcutaneous injection, usually in the upper arm)	Did the child receive an injection in the arm against [use local term for measles], after he/she was old enough to sit up or crawl?

DTP, diphtheria toxoid, tetanus toxoid, and whole cell pertussis vaccine combination; OPV, oral polio vaccine.

**Table 4:** World Health Organization–recommended EPI schedule, 2012.

Age of Infant	Parenteral Vaccines	Oral Vaccines
Birth	BCG, HBV^a^	OPV^b^
6 weeks (some countries give this dose at 8 weeks)	DTP, Hib, HBV, usually administered as pentavalent combination^a^; 10- or 13-valent PnCV^c^	OPV; rotavirus vaccine (Rotateq or Rotarix)
10 weeks (some countries give this dose at 16 weeks)	Pentavalent combination^a^; 10- or 13-valent PnCV (3p+0 schedule)^c^	OPV; rotavirus vaccine (Rotateq or Rotarix)
14 weeks (some countries give this dose at 24 weeks)	Pentavalent combination^a^; 10- or 13-valent PnCV^c^	OPV; rotavirus vaccine^d^ (Rotateq)
9–12 months	Measles^e^ (rubella^f^, with measles); 10- or 13-valent PnCV (2p+1 schedule)^c^; yellow fever (endemic countries)^g^; Japanese encephalitis (endemic countries)^h^	

Adapted from [67].

a Since perinatal or early postnatal transmission is an important cause of chronic infections globally, all infants should receive their first dose of hepatitis B vaccine as soon as possible (<24 hours) after birth even in low-endemicity countries. The primary hepatitis B immunization series conventionally consists of three doses of vaccine (one monovalent birth dose followed by two monovalent or combined vaccine doses at the time of DTP1 and DTP3 vaccine doses). However, four doses may be given for programmatic reasons (e.g., one monovalent birth dose followed by three monovalent or combined vaccine doses with DTP vaccine doses), according to the schedules of national routine immunization programs.

b OPV alone, including a birth dose, is recommended in all polio-endemic countries and those at high risk for importation and subsequent spread. A birth dose is not considered necessary in countries where the risk of polio virus transmission is low, even if the potential for importation is high/very high.

c For infants, three primary doses (the 3p+0 schedule) or, as an alternative, two primary doses plus a booster (the 2p+1 schedule). If the 3p+0 schedule is used, vaccination can be initiated as early as 6 weeks of age with an interval between doses of 4–8 weeks. If the 2p+1 schedule is selected, the two primary doses should ideally be completed by 6 months of age, starting as early as 6 weeks of age with a minimum interval of 8 weeks between the two doses (for infants aged ≥7 months a minimum interval of 4 weeks between doses is possible). One booster dose should be given at 9–15 months of age.

d If Rotarix is used, only two doses are administered.

e In countries that have achieved a high level of control of measles, the initial dose of measles vaccine can be administered at 12 months of age. All children are currently expected to receive a second dose of measles vaccine. In the least developed countries this is often administered through mass immunization campaigns.

f Rubella vaccine, administered in combination with measles vaccine, is recommended for countries that reliably administer two doses of measles vaccine and have achieved a high level of measles control.

g Yellow fever should be co-administered at the infant visit when measles vaccine is administered.

h Japanese encephalitis vaccines may be given at age 12 months for children living in highly endemic areas.

DTP, diphtheria toxoid, tetanus toxoid, and whole cell pertussis vaccine combination; HBV, hepatitis B vaccine; Hib, *Haemophilus Influenzae* type b conjugate vaccine; OPV, oral polio vaccine; pentavalent combination, DTP+HBV+Hib formulated to be administered in combination as a single injection; PnCV, pneumococcal conjugate vaccine containing either 10 or 13 separate conjugates of different capsular serotypes.

## Data Analysis and Reporting Issues

Traditionally, surveys report the proportion of persons who have been vaccinated as recorded “by card only” and by “card plus history,” both by age 12 months and by age at the time of the survey. EPI surveys also calculate and report separately on coverage of “valid” doses among children with cards, such as a minimum interval of 28 days between doses of diphtheria-tetanus-pertussis-containing vaccines and a minimum age of 270 days for measles vaccination [21]. As coverage increases, evaluation of the timeliness of vaccination among children with documented dates of birth, and of each vaccine dose, provides additional information to guide program performance. Timeliness can be illustrated through graphs of the distribution of age at receipt of each dose compared to the national schedule [32],[38] or by time-to-event curves of the cumulative coverage by age [32],[39]. The mean number of extra days or weeks that children remain under-vaccinated and at risk of disease [38],[40],[41] and risk factors for delay in vaccination can be assessed [39],[40].

Surveys that use probability proportional to estimated size sampling without stratification assume that each cluster has equal weight in the analysis. EPI surveys do not collect data on the number of eligible households in each cluster, and cannot validate this assumption. Consequently, if outdated or inaccurate sampling frames are used, the resulting point estimate may be biased. If, however, a household listing step is included in the survey preparation and sampling stages, appropriate weights can be calculated and used to derive national estimates and confidence intervals, as is recommended nowadays by DHS and MICS protocols [18].

The standard error of the coverage estimate is traditionally used to calculate and report a 95% confidence interval around the point estimate. The confidence interval is affected by the sample size, the sampling design, and the underlying proportion itself. Because individuals living in one cluster of a population tend to be more similar to each other than persons from different clusters, respondents in a cluster sample each contribute less independent information about the overall population than respondents in a simple random sample. This positive intra-cluster correlation causes cluster samples to have a wider confidence interval around the point estimate of the population parameter than a simple random sample of the same size. DHS, MICS, and EPI surveys all provide guidance on estimation of confidence intervals for key indicators, but the degree to which confidence intervals are reported and used varies widely, as discussed elsewhere in this Collection [42].

The application of standard statistical techniques to estimate confidence intervals has been challenged for surveys that use non-probability sampling of households within each cluster [43], although simulations of results from EPI surveys have shown that confidence intervals in these surveys are generally within the desired precision of ±10 percentage points [44]. Some variations on the EPI survey method take a probability sample (e.g., a systematic random sample in the final stage) [22]–[26], which makes it possible to calculate sampling weights and construct meaningful confidence intervals.

**Figure 3:**
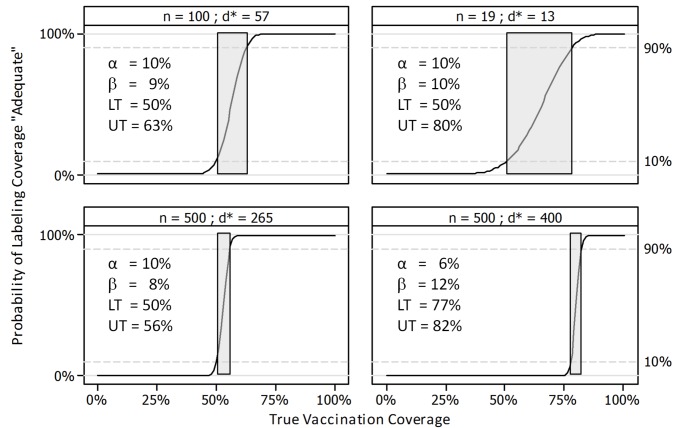
Operating characteristic curves for four LQAS sampling plans. In each panel, the curve indicates the probability of finding d* or more vaccinated children in a random sample of size *n*. Lots with coverage≤lower threshold (LT) will be classified as having inadequate coverage with probability ≥(1 − α). Lots with coverage≥upper threshold (UT) will be classified as having adequate coverage with probability ≥(1 − β). The gray area is the region where LT<coverage<UT; lots with coverage in the gray area may be labeled either adequate or inadequate. The gray area includes the region of coverage, for instance, where there is a 50/50 probability of being classified adequate or inadequate. Neither classification (adequate or inadequate) rules out the strong possibility that the true coverage lies in the gray area. The gray area may be made larger or smaller and may be moved to regions of higher or lower coverage by manipulating LT, UT, α, and β to arrive at different values of *n* and d*.

LQAS surveys inevitably have a central range of coverage (the gray area) that is not excluded by either the “adequate” or the “inadequate” classification. That is, neither classification excludes the medium category. For fixed values of alpha and beta (the probability of type I and II errors, respectively), a larger sample size per lot will result in a narrower gray area and a correspondingly more confident conclusion about whether coverage is likely to be adequate (Figure 3). When data are combined across numerous lots, it is possible to estimate a region-wide proportion and confidence interval using formulae from stratified sampling and applying strata and cluster weights. However, at the level of the individual lot, the user does not obtain a precise coverage estimate from a LQAS survey, but only an assurance that coverage in populations where there is very low coverage is very likely to be classified as inadequate and that coverage in populations where there is very high coverage is very likely to be classified as adequate.

In the analysis phase, survey analyses are usually restricted to respondents with complete data. However, analyses of DHS and MICS surveys prior to 2002 showed that maternally recalled vaccination data were internally consistent, and that inclusion of a verbal history of vaccination in results was preferable to other options such as restricting analyses to children with HBRs or assuming that coverage among those without HBRs was the same as those with HBRs [45]. Inclusion of children without HBRs is only possible for calculation of percentage coverage, however, and not for assessing timeliness of vaccination.

When measuring vaccination coverage from survey data, if data are missing for reasons related to the likelihood of vaccination, restricting analyses to those with complete data will bias results. Analysts can impute hypothetical values for missing responses, but imputing a single value fails to account correctly for the uncertainty associated with selecting an arbitrary (though perhaps plausible) value to impute. More sophisticated methods include integrating over a likelihood function, or imputing numerous values for each missing datapoint (multiple imputation), and are preferred but rare in vaccination coverage measurement [46],[47]. DHS and MICS surveys adjust sampling weights for nonresponse, and may impute a single value when a HBR vaccination date is invalid (e.g., lists day 32 of the month) or missing, but do not currently employ multiple imputation in vaccination coverage estimates [48], although this could be introduced in the future as the technique of multiple imputation becomes more accessible.

## How Can the Challenges Facing the Measurement of Vaccination Coverage Be Addressed?

Vaccination coverage is an important indicator that is used to monitor not only immunization programs but also health system performance at national and global levels. Coverage surveys can include questions on reasons for receiving, or not receiving, vaccines, and investigate demographic and other factors associated with coverage [49]. In addition, in the specific context of measles vaccination, coverage is a key component of the cohort analyses that estimate the build-up of susceptible children after vaccination campaigns and that identify when follow-up campaigns are needed [50]. Inflated coverage data contribute to delays in implementing follow-up campaigns, which may lead to measles outbreaks [51]. Finally, coverage data can also be used in field evaluation of vaccine effectiveness [52].

Despite these important public health applications of vaccination coverage data, many challenges face the collection of accurate coverage data [5],[6] that must be addressed to guide program implementation and to ensure that funding decisions are based on real performance. For example, although survey data are often preferred to administrative reports for the reasons that we outlined at the start of this article, surveys are also subject to errors (Table 2). Groups that may be omitted from sampling frames—for example, those living in conflict-affected or otherwise inaccessible areas or in rapidly growing urban areas [53], and recent migrants—may have lower vaccination coverage [1]. Depending on the size of these population groups and the purposes of the evaluation, substantial extra effort may be needed to update the sampling frame or to conduct special surveys among these groups.

Among potential sources of non-sampling error, one of the most critical challenges is the availability of HBRs, which has been low in surveys of some of the countries contributing most to global estimates of vaccination coverage [54]. In India, WHO and UNICEF coverage estimates since 2009 have been based on a 2008 coverage evaluation survey in which vaccine cards were seen for only 52% of children [55]; in Nigeria, HBRs were seen in 40% of children in the 2009 national immunization coverage survey and in only 26% in the 2007 DHS [56]. Obtaining records of vaccines administered after infancy, including booster doses and vaccines targeted to older age groups such as tetanus toxoid, which is administered to pregnant women, is even more difficult. Records of administration of vaccines containing tetanus toxoid may be available for the most recent pregnancy but not for previous pregnancies, mass campaigns, or early childhood. Serological surveys show that vaccination coverage surveys tend to underestimate the prevalence of protection against neonatal tetanus [57]. Thus, unless primary recording of vaccination data on HBRs and clinic records is improved, investment in other strategies to improve survey methods or administrative estimates will have limited effect.

When choosing a survey methodology, decision makers should consider both the specific information needed and the speed with which the information is required. The timing and frequency of DHS and MICS surveys are not decided by immunization program managers, and thus data are used by these health professionals as and when they become available (which is several months after survey completion even for the preliminary results). In the years between DHS and MICS surveys, program managers may need additional information on vaccination coverage to improve planning and may prefer the quicker and less expensive EPI surveys; we propose ways to improve these below. Sometimes, vaccination coverage estimates are needed very rapidly (e.g., during campaigns, while the vaccination team is still in the area), and a purposive sampling method focused on areas most likely to have low coverage has been used [9],[58]. Recent experience with rapid monitoring of polio campaigns using purposive sampling, however, has not been positive, and LQAS surveys are now promoted for polio campaign monitoring [31].

Although biomarkers are under consideration for validating coverage, they have several limitations for this purpose and are not currently included routinely in surveys measuring childhood vaccination coverage. For most vaccines, the presence of antibody following vaccination cannot be distinguished from that following “natural” infection. Exceptions are the presence of tetanus antibody (infection does not generate lasting immunity) and the presence of antibodies to antigens included in subunit vaccines such as hepatitis B but not to other antigens found in the whole organism (an indication of vaccine-induced immunity) [59]. Even for these vaccines, detection of antibodies does not indicate reliably how many doses have been received [60]. Furthermore, absence of antibody means either that the child is unvaccinated or that vaccines have lost their potency. Biomarkers are therefore potentially useful to estimate population-level protection [61] but not necessarily to validate coverage measurements or vaccination program performance. The development of antibody assays on oral fluid samples for tetanus [60] and measles [62] may make surveys with repeated sample collection more acceptable and may allow evaluation of vaccination campaigns [63].

## Recommendations and Conclusions

To reduce bias in coverage measurement by any method, we recommend that primary recording of vaccination data be improved. In the long term, we recommend further investment in the development, evaluation, and roll-out of effective systems for digital recording and data transmission. In the short-to-medium term in low- and middle-income countries, paper-based recording must be improved. Record design will need to evolve rapidly to accommodate the introduction of new and underutilized vaccines and to allow recording of doses administered through campaigns and across the life course. We recommend that research be conducted to improve the design of primary records to reduce recording errors and facilitate compilation of data. We also recommend that health workers educate mothers so that they value HBRs, understand the information therein, and retain them safely.

Whenever surveys are conducted, all efforts to ensure high-quality data should be made (Table 2). In particular, probability samples should be used, the sample size and number of clusters should be selected appropriately for the survey objectives, efforts should be made to encourage mothers to have HBRs ready at the time of the survey, households should be revisited if necessary to interview a suitable respondent and see the HBR, health facilities should be visited to look for records of children whose HBRs were unavailable, and strict quality control measures should be implemented. Analyses should incorporate internal consistency checks as part of quality control, including assessment of the validity of the verbal history of vaccination. These checks include comparison of prevalence of a Bacille Calmette Guerin vaccine against tuberculosis (BCG) scar among children with documented versus verbal history of BCG [32], and comparison of coverage among those who reported receiving a vaccination card but did not present it and those who presented a card [45]. In multi-indicator surveys, vaccination coverage could be cross-tabulated against coverage of other interventions for persons with and without a HBR; if the verbal history is reliable, the same associations should be found in both groups. Large-scale survey programs should also be evaluated periodically to sustain attention to quality control [64].

Technical expertise for collecting high-quality data and interpreting and using results needs to be further developed at national and sub-national levels. Program managers need to use coverage data with other program indicators to improve program planning and management. Identification of low-coverage areas should trigger action to reach underserved children, who are often those at highest risk of dying should they acquire a vaccine-preventable infection [65]. As a measure of population protection, coverage is currently limited by assumptions about vaccine effectiveness and thus is helpful but not sufficient. Additional information from vaccine management assessments, surveillance, outbreak investigations, and, where available, special studies such as case control studies of vaccine effectiveness should be reviewed together with coverage data to obtain a fuller picture of program success. Sero-surveys and vaccination coverage surveys are likely to complement each other for the foreseeable future, and we recommend that further research into the use of sero-surveys and the development of new biomarkers be undertaken.

Sources of uncertainty in surveys must be considered before drawing strong conclusions about their results. It is common to focus on the point estimate of coverage from the survey, but it is also important to consider uncertainty due to sampling design, which is usually expressed using a confidence interval, and potential biases, which should be assessed by reviewing information about the survey protocol and its implementation. The recent inclusion of a “grade of confidence” in national immunization coverage estimates produced by WHO-UNICEF is an important first step towards improving the usefulness of these estimates, and we recommend that grading of the quality of surveys should also be done. In particular, when LQAS surveys are done, we recommend that their results be interpreted with due recognition of the gray area [66].

Finally, as we mentioned at the start of this review, there is currently tension between financing systems, which reward high coverage, and efforts to improve the quality of coverage measurement. We believe and strongly recommend that it is time to reward actions that improve the quality of data, particularly those discussed in this review, rather than rewarding a country's apparent coverage achievements.

**Key Points**

- Vaccination coverage is an important indicator of public health if measured accurately; at present, well-designed and executed surveys provide more accurate and comparable results than administrative reports, which are subject to incomplete and inaccurate reporting of the numerator and inaccurate estimation of the denominator.
- To reduce bias in coverage measurement based on surveys and on administrative reports, primary recording of vaccination data on home-based records and clinic records must be improved. In the long term, this will involve digital recording and data transmission. In the short-to-medium term in low-income countries, paper-based recording must be improved.
- Whenever surveys are done, to minimize selection bias and information bias, the sample size should be selected according to program needs, probability sampling should be used, and strict quality control measures should be implemented for data collection and analysis.
- The potential magnitude of bias in surveys must be assessed before results are interpreted, quality assessment criteria should be developed and endorsed by partners, and partners should consider uncertainty in coverage estimates before basing decisions such as those involving performance-based financing on coverage.
- To improve program performance, national immunization programs and their partners should take action to improve the collection, interpretation, and use of vaccination coverage data together with data on other indicators.

## Abbreviations

BCG: Bacille Calmette Guerin vaccine against tuberculosis
DHS: Demographic and Health Survey/s
EPI: Expanded Programme on Immunization
HBR: home-based record
LQAS: Lot Quality Assurance Sampling
MICS: Multiple Indicator Cluster Survey/s
UNICEF: United Nations Children's Fund
WHO: World Health Organization
